# Halotolerant aminopeptidase M29 from *Mesorhizobium* SEMIA 3007 with biotechnological potential and its impact on biofilm synthesis

**DOI:** 10.1038/s41598-017-10932-8

**Published:** 2017-09-06

**Authors:** Elwi Machado Sierra, Mariana Rangel Pereira, Thaís Carvalho Maester, Elisangela Soares Gomes-Pepe, Elkin Rodas Mendoza, Eliana G. de Macedo Lemos

**Affiliations:** 1 Department of Technology, São Paulo State University, Jaboticabal, São Paulo State, Brazil; 2 Institute for Research in Bioenergy (IPBEN), Jaboticabal, São Paulo State Brazil; 3 Universidad Simón Bolívar, Barranquilla, Colombia; 4 Av. Prof. Paulo Donato Castellane, s/n. Jaboticabal, Post code 14884-900 São Paulo State, Brazil

## Abstract

The aminopeptidase gene from *Mesorhizobium* SEMIA3007 was cloned and overexpressed in *Escherichia coli*. The enzyme called MesoAmp exhibited optimum activity at pH 8.5 and 45 °C and was strongly activated by Co^2+^ and Mn^2+^. Under these reaction conditions, the enzyme displayed K_m_ and k_cat_ values of 0.2364 ± 0.018 mM and 712.1 ± 88.12 s^−1^, respectively. Additionally, the enzyme showed remarkable stability in organic solvents and was active at high concentrations of NaCl, suggesting that the enzyme might be suitable for use in biotechnology. MesoAmp is responsible for 40% of the organism’s aminopeptidase activity. However, the enzyme’s absence does not affect bacterial growth in synthetic broth, although it interfered with biofilm synthesis and osmoregulation. To the best of our knowledge, this report describes the first detailed characterization of aminopeptidase from *Mesorhizobium* and suggests its importance in biofilm formation and osmotic stress tolerance. In summary, this work lays the foundation for potential biotechnological applications and/or the development of environmentally friendly technologies and describes the first solvent- and halo-tolerant aminopeptidases identified from the *Mesorhizobium* genus and its importance in bacterial metabolism.

## Introduction

A large diversity of *Mesorhizobium* species have been identified in different environmental niches, including artic soil^1^, marine sponge^2^, semi-arid and saline soil^3^, and polychlorinated biphenyl (PCB) contaminated soil^4^. The large distribution of *Mesorhizobium* sp. suggests that species within this genus have adapted to several eco-climatic conditions because of its heterogeneous and extremely plastic genome. Although knowledge of the role of *Mesorhizobium* in the nitrogen-fixing process is currently expanding, the potential for its use in biotechnology is still limited, because only a few biomolecules with industrial application have been reported. These enzymes with industrial applications include cyclic β-(1,2)-glucans^5^, L-ribulose 3-epimerase^6^, β-transaminase^7^, cellulase^8^, L-rhamnose isomerase^9^ and pyridoxine 4-oxidase^10^. However, despite the potential industrial relevance and a large database of peptidases [3179 known peptidases, and homologues from 29 *Mesorhizobium* genomes], there is almost no information concerning the biochemistry of the proteolytic system or any peptidase from the *Mesorhizobium* genus.

Aminopeptidases (EC 3.4.11) are a diverse group of exopeptidases involved in the catalysis of peptide bond cleavage at the amino-terminus of proteins, releasing amino acids residues, preferably hydrophobic ones. These enzymes are widely distributed in bacteria, fungi, plants and animal tissues with important physiological roles, such as the maturation and degradation of proteins, the regulation of hormonal levels, defence control, hydrolysis of regulatory peptides, regulation of genes expression, support of the amino acids pool^11^, peptidoglycane metabolism^12^, nitrogen nutrition and virulence factors^13^. Aminopeptidases are used extensively in the biopharmaceutical industry to remove N-terminal sequences in the production of anti-hypertensive peptides^11^ and antiviral compounds^14^. Additionally, these enzymes have an important role in the food industry by modifying organoleptic characteristics (e.g., texture, bitterness and flavour)^15^. Furthermore, aminopeptidases can be used with other endopeptidases for wastewater treatment to degrade proteins^16^.

Over the last years, several types of experiments to improve the physicochemical characteristics of these enzymes have been performed including chemical modification, protein immobilization, protein engineering and directed enzyme evolution. However, if the enzymes are naturally stable and exhibit high activities in the presence of physicochemical factors, such modifications are not necessary. Therefore, it is conceivable that genomic data mining could be used to find an enzyme naturally stable or catalytically efficient enough to optimally fit process requirements that are solely dictated by substrate and product properties. In the era of big data, the genomic resources in various databases are highly valuable, making genomic data mining a growing area with an unprecedented capacity for the discovery of novel enzymes, since a huge abundance of enzymes already exists in these unexplored genomic resources.

In this study, we report the discovery of the *mesoamp* gene by genomic data mining. The gene shares 54% sequence identity with aminopeptidase T from *Thermus thermophilus* when analysed with The Peptidase Database (MEROPS). This gene was cloned into an expression vector and the recombinant protein, named MesoAmp, was functionally and physically characterized. Our results show that MesoAmp could be overexpressed in a stable dimeric form and purified without contaminants. The functional characterization of the enzyme showed that it was active in the presence of a high concentration of salt and organic solvents. Taken together, these data demonstrate the interesting features of MesoAmp in bacterial metabolism and support the potential of this enzyme for use in biotechnological processes.

## Results

### Sixty-eight peptidases were predicted by comparative genomic analysis

The automatic annotation tool identified 101 ORFs that possibly encode peptidases that are similar to the *Mesorhizobium* genome annotated in MEROPS^17^. To characterize the proteolytic machinery of the *Mesorhizobium* genus, the protein sequences of each of the 101 ORFs were subjected to an exhaustive search against the MEROPS peptidase database. Using the comparative database search, we detected a total of sixty-eight peptidases belonging to 26 clans of proteolytic enzymes. These enzymes were distributed into groups based on the catalytic type of the proteolytic mechanism: 6.8% aspartic, 3% glutamic, 2.7% cysteine, 41.1% metallo, 35.6% serine, 4.1% treonin, 4.1% mixed and 5.5% peptidase inhibitors (Table S2). This finding supports our hypothesis that a large number of peptidases with biotechnological potential remain unexplored in the *Mesorhizobium* genome.

### Three peptidases with biotechnological potential were discovered

Considering the physicochemical characteristics of peptidases with similar sequences and biochemical characteristics as pH, temperature and thermostability, we identified three enzymes with potential biotechnological applications (Table S3).

We selected Aminopeptidase T and called the enzyme MesoAmp. We focused on this enzyme for further bioinformatic analysis and biochemical characterization.

### Sequence analysis of MesoAmp revealed the amino acid fingerprint of thermophilic metalloproteases

The sequence of MesoAmp consists of 1257 pb (G + C content = 64%) encoding a protein with 418 amino acids. No signal peptide sequence was identified. The comparison of the complete amino acid sequence with the proteins from the database displayed the highest homology (95%) with the aminopeptidase from *Mesorhizobium ciceri* and another species of *Mesorhizobium*. MesoAmp has 54% sequence identity with an aminopeptidase T (AmpT) from *Thermus thermophilus* (MER001285), 40% sequence identity with aminopeptidase S (PepS) (MER005731) and the aminopeptidase S (AmpS) (MER014416) enzymes from *Streptococcus thermophilus* and *Staphylococcus aureus*, respectively.

The multiple sequence alignment of the MesoAmp sequence with AmpT, PepS and AmpS revealed an 8 amino acid fingerprint that provides a signature for a thermophilic metalloprotease^18^, as well as the catalytic amino acid Tyr at position 352 and the metal-binding residues Glu259, Glu325, His354, His387 that are shown in detail in Supplementary Figure S1. The analysis using the ConSurf server revealed that the most conserved amino acids located in the C-terminus of the protein (Supplementary Figure S1).

### Dimeric structure of MesoAmp with high degree of conservation

**Figure 1:**
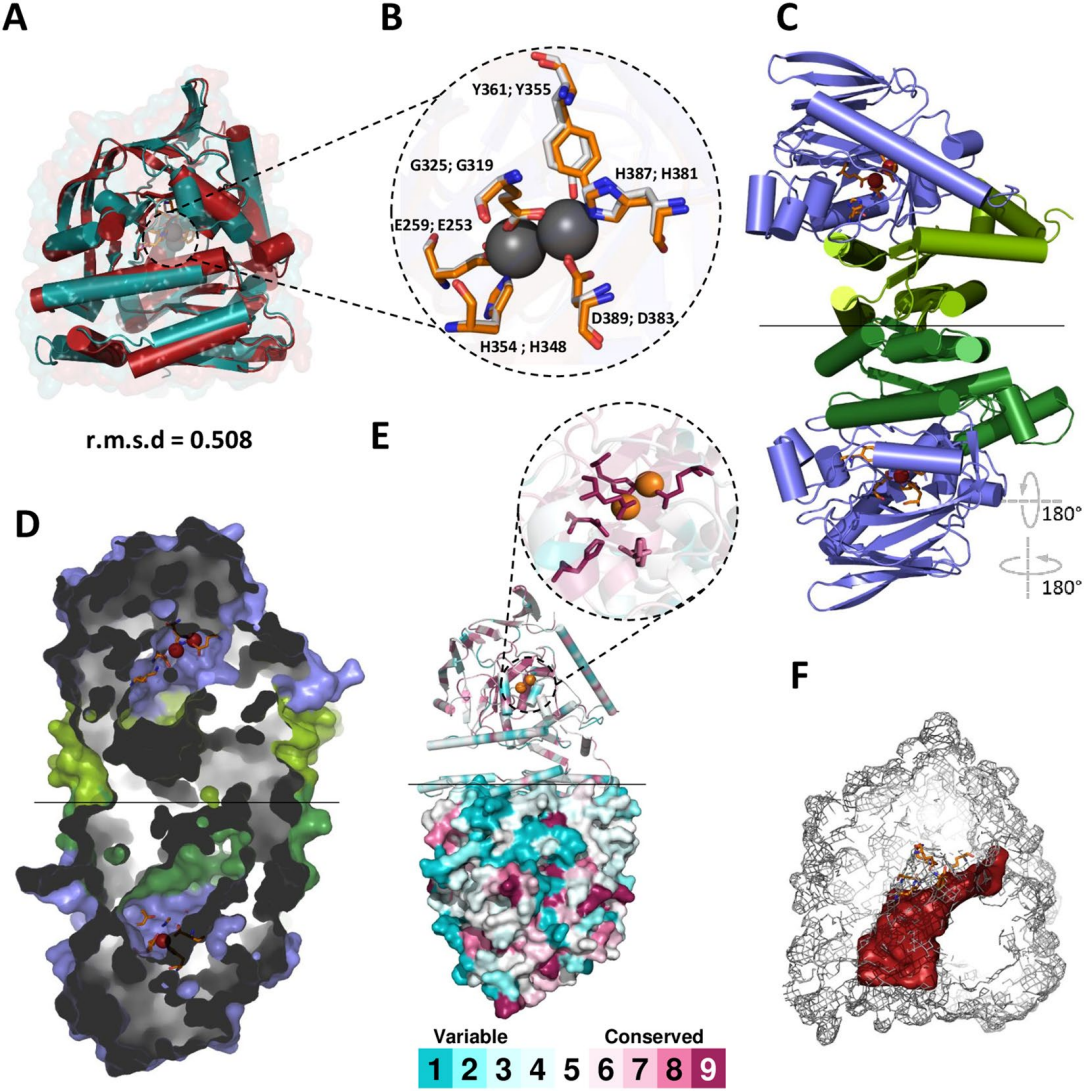
The structural features of the MesoAmp model. (**A**) The superimposed structures of MesoAmp model (gray) with the structure of *Staphylococcus aureus* aminopeptidase chain A (blue), revealing the conservation of the alpha/beta fold, with a root mean square deviation (r.m.s.d) of 0.582 Å. The metal atoms are shown as gray spheres. (**B**) The superimposition of the catalytic residue (Tyr 361) and the metal-binding residues Glu259, Glu325, His354, His387 and Asp389, and their counterpart residues in the AmpS structure and the cobalt atoms are shown as gray spheres. (**C**) A cartoon structure of the dimeric arrangement of MesoAmp with its N-terminal domain involved in dimerization shown in green and its C-terminal domain (catalytic region) shown in blue. (**D**) Modelling representation of the substrate-binding site of MesoAmp homodimer using the code-color conservation of Consurf server. (**E**) The mesh representation of the substrate-binding hole (gray) and binding pocket (red) with substrate Trp-Gly (WG). r.s.m.d = root-mean-square deviation.

The three-dimensional model of MesoAmp was created using Modeller and validated using ModFold that demonstrated that there was less than a 1:1,000 chance that the models were incorrect. The Ramachandran plots for the models revealed a relatively high number of amino acids in allowed regions (98.08%) that is an indication for further model optimizations. The structural model of MesoAmp superimposed remarkably well with the AmpS structure, displaying a r.m.s.d of 0.508 Å (Fig. 1A and B). The analysis using Z-Dock revealed a homodimer structure with an N-terminal dimerization domain (Fig. 1C). The conserved residues between scarcely conserved clusters located on the surface of the protein (Fig. 1D) are presumably important for the maintenance of the structural integrity of these surface patches^19^. Buried inside the protein is the highly conserved region constituting the substrate–binding pocket (Fig. 1D and E) and the substrate-binding cavity of 1,780 Å^3^ (Fig. 1F).

### MesoAmp was expressed in the soluble fraction and the active enzyme obtained was in a dimeric form

The optimal conditions for the production of the soluble and active form of MesoAmp from *E. coli* BL21(DE3) were IPTG at a final concentration of 0.1 mM, an induction temperature of 30 °C and 12 h growth under 200 rpm. Under these conditions, the recombinant protein was soluble in the supernatant of the cell lysate that aided further purification. The SDS–PAGE analysis of purified MesoAmp revealed the enzyme was completely purified by affinity chromatography and weighed 45.72 kDa under denaturing conditions (Supplementary Figure S2A) as predicted with ProtParam tool (http://us.expasy.org/tools/protparam.html). The molecular mass of the native enzyme was estimated to be 88.05 kDa by gel filtration (Supplementary Figure S2B). Our results indicate that the enzyme is a homodimer that is highly common for the aminopeptidase M29 family^20^.

### MesoAmp is active in a wide range of pH values and temperatures

**Figure 2:**
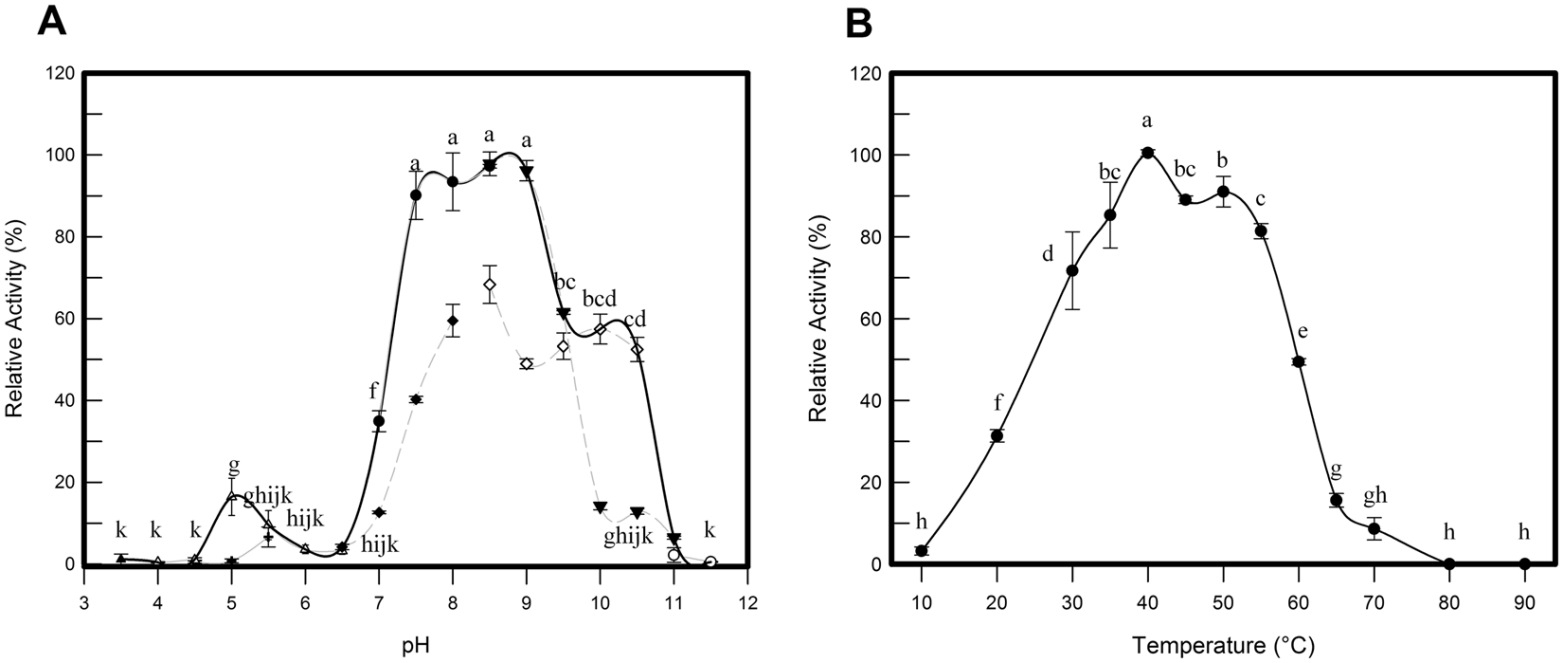
Physicochemical analysis of MesoAmp. (**A**) The pH effect on the enzymatic activity of MesoAmp in different buffers: McIlvaine buffer (▴); Sodium citrate buffer (∆); Sodium phosphate buffer (♦); Tris-HCl buffer (•); Bicarbonate-Sodium hydroxide buffer ▾); Glycine-sodium hydroxide buffer (◊) and Monosodium phosphate - Sodium hydroxide buffer (○). (**B**) The temperature effect on MesoAmp activity determined in 100 mM Bicarbonate-Sodium hydroxide buffer (pH 8.5). The data are expressed as Mean ± SD of five independent experiments.

The effects of pH and temperature on MesoAmp activity were examined spectrophotometrically using Leu-p-NA as substrate. The pH curve displayed a maximum activity within a narrow pH range from pH 7.5 to 9.0 with no significant difference between these pH values (Fig. 2A). The enzymatic activity was almost completely lost when the pH was lower than 6.5 or higher than 10.0. The experiments subsequently described were carried out in 100 mM bicarbonate sodium-hydroxide buffer at pH 8.5. The purified recombinant MesoAmp exhibited the highest activity at 40 °C. Interestingly, MesoAmp still maintained more than 80% of its activity in the temperature range from 35 to 55 °C (Fig. 2B).

The CD spectrum of the recombinant protein displayed two negative peaks in the far-UV region at 208 nm and 222 nm, as expected for alpha-beta secondary structure (Supplementary Figure S3A). MesoAmp displayed a melting temperature (T_m_) of 53.2 °C in the presence and absence of Co^2+^ ions. Therefore, the cobalt ion does not change the alpha-beta content in the thermal denaturation assay (data not shown). Temperatures above 40 °C induced the denaturation of the α-helical structure of MesoAmp, showing a transition to the irreversible state (Supplementary Figure S3B). The disruption of its secondary structure begins below 55 °C as indicated by the increase in the unfolded fraction (60% unfolded), whereas the enzymatic activity is not significantly altered up to this point.

Metallopeptidases exhibit a broad range of metal-ion dependencies. The activity of MesoAmp in the presence of different metal ions is shown in Table 1. Approximately 90% of the activity was lost in the presence of Ca^2+^, Ba^2+^, Al^2+^ and Zn^2+^ ions. Conversely, MesoAmp displayed enhanced catalytic activity in the presence of Co^2+^ and Mn^2+^ ions (17-fold and 8-fold, respectively). All other metal ions tested (with a rank order of Mg^2+^ > Ni^2+^ > Mg^2+^ > I^2+^) had stimulatory effects on the enzymatic activity of MesoAmp.

**Table 1:** The effect of various divalent cations on the MesoAmp activity.

Metal ion		relative activity (%)	
None		100	ghij
Al^2+^		5.43 ± 0.84	k
Ba^2+^		19.50 ± 0.97	ijk
Ca^2+^		7.36 ± 0.71	k
Cd^2+^		13.93 ± 2.76	jk
Mg^2+^		152.07 ± 5.28	gh
Zn^2+^		11.29 ± 2.64	jk
Cu^2+^		65.21 ± 9.81	hijk
I^2+^		108.50 ± 6.37	ghi
Li^2+^		76.78 ± 3.48	hijk
Ni^2+^		179.00 ± 6.29	fg
Mn^2+^			
	1 mM	844.01 ± 77.52	c
	2 mM	284.29 ± 26.63	e
	3 mM	295.93 ± 49.85	e
	4 mM	334.36 ± 26.71	e
	5 mM	473.02 ± 41.11	d
Co^2+^			
	1 mM	51.36 ± 13.95	ijk
	2 mM	412.86 ± 63.63	d
	3 mM	1460.93 ± 88.51	b
	4 mM	1768.09 ± 133.65	a
	5 mM	1597.07 ± 59.58	b

The small letters on the right of the values (a, b, c,… k) indicates the significant difference between each condition tested in the experiment, according to an ANOVA and Tukey’s test at 5% probability. The data are expressed as Mean ± SD of seven independent experiments.

After determining the optimum temperature and pH, the activity of MesoAmp was tested with Leu-p-NA at pH 8.5, 40 °C in the presence of 3 mM Co^2+^. The K_m_, V_max_ and K_cat_ values were calculated to be 0.2364 ± 0.0182 mM, 1.115 ± 0.020 mM.s^−1^ and 712.1 ± 88.12 s^−1^ respectively.

Thermal treatment at 45 and 50 °C after 60 min pre-incubation did not influence the activity of the enzyme. However, MesoAmp lost half of its activity when incubated at 55 °C for 15 min, and it was rapidly inactivated when incubated at 70 °C (Supplementary Figure S4). After 5 min incubations at these higher temperatures, the enzymatic activity decreased by approximately 95%.

### MesoAmp retains its activity in the presence of organic solvents and at high salt concentrations

PMSF and iodoacetic acid did not influence enzyme activity. However, metal-chelating agents such as EDTA severely inhibited its activity (Table 2) indicating that MesoAmp is a metallopeptidase. We tested the activity of the enzyme in the presence of denaturing agents (Table 2). The MesoAmp activity was very sensitive to concentrations of guanidine-HCl between 0.5 and 2.0 M, indicating a significant disruption in the secondary structure of the protein. However, urea treatment did not substantially affect the activity at concentrations of 0.5 M and 1 M but reduced the activity by half when at 2 M.

**Table 2:** The effect of inhibitors and denaturing agents on the activity of MesoAmp*.

Inhibitors		Relative activity (%)	
None		100	bc
EDTA			
	5 mM	48.76 ± 3.39	ij
	10 mM	32.29 ± 0.56	kl
	20 mM	18.41 ± 0.76	mn
PMSF 10 mM		96.89 ± 1.75	bc
Iodoacetic acid 10mM		97.93 ± 2.89	bc
**Denaturant agent**		**Relative activity (%)**	
None		100	bc
β-Mercaptoethanol			
	1 mM	80.16 ± 4.18	de
	5 mM	61.67 ± 1.22	jk
	10 mM	32.29 ± 0.56	lm
DTT			
	1 mM	10.07 ± 0.93	no
	5 mM	2.12 ± 0.07	o
	10 mM	1.41 ± 0.06	o
Guanidine-HCl			
	0.5 M	67.78 ± 0.40	fg
	1 M	29.10 ± 1.11	lm
	2 M	3.50 ± 0.34	o
Urea			
	0.5 M	92.65 ± 7.78	bc
	1 M	73.36 ± 6.25	ef
	2 M	54.11 ± 3.27	hi
NaCl			
	0.5 M	127.31 ± 9.11	a
	1 M	125.94 ± 3.49	a
	2 M	121.81 ± 0.16	a
	3M	103.45 ± 2.76	bc
	4M	87.54 ± 1.56	ef

*Enzyme assays were performed in 100 mM Bicarbonate-Sodium hydroxide buffer at pH 8.5 and 40 °C.

The small letters on the right of the values (a, b, c,…m) indicates the significant difference between each condition tested in the experiment, according to an ANOVA and Tukey’s test at 5% probability. The data are expressed as Mean ± SD of seven independent experiments.

Zhuo and Dixon^21^ found that the role of sulfhydryl reagents was to chelate small amounts of metal ions. To prevent this chelating mechanism, we measured the enzymatic activity without metal ions. The role of DTT as a metal chelator makes it unsuitable to use with MesoAmp. As shown in Table 2, the presence of DTT and β-mercaptoethanol considerably decreased the enzymatic activity. On the other hand, NaCl treatment enhanced the activity of MesoAmp (Table 2) by 1.2-fold compared to the enzymatic activity without NaCl.

The presence of non-ionic surfactants, such as Tween (20 and 80) and Triton (X-100 and X-114), slightly decreased the amount of enzymatic activity (Table 3). Nevertheless, ionic and cationic surfactants (SDS and CTAB) resulted in complete loss of MesoAmp activity (data not shown). Previous reports have indicated that the inhibitory effect of SDS or CTAB could be caused by their binding to the enzyme at more than one site, thus modifying the tertiary structure and altering the charge distribution^20^.

**Table 3:** The effect of various detergents on the activity of MesoAmp*.

Detergents		Relative Activity (%)	
None		100	bc
Triton X100			
	0.5%	76,81±3,236	fghi
	1%	73,74±5,970	ghi
	2%	70,15±1,688	i
Triton X114			
	0.5%	81,64±4,532	efgh
	1%	75,89±4,341	fghi
	2%	83,47±1,751	defg
Tween 20			
	0.5%	73,77±1,840	ghi
	1%	71,20±2,962	hi
	2%	69,54±2,090	i
Tween 80			
	0.5%	77,24±3,491	fghi
	1%	84,43±5,220	defg
	2%	90,25±2,714	cde

*Enzyme assays were performed in 100 mM Bicarbonate-Sodium hydroxide buffer at pH 8.5 and 40 °C. The small letters on the right of the values (a, b, c,…i) indicates the significant difference between each condition tested in the experiment, according to an ANOVA and Tukey’s test at 5% probability. The data are expressed as Mean ± SD of seven independent experiments.

**Figure 3:**
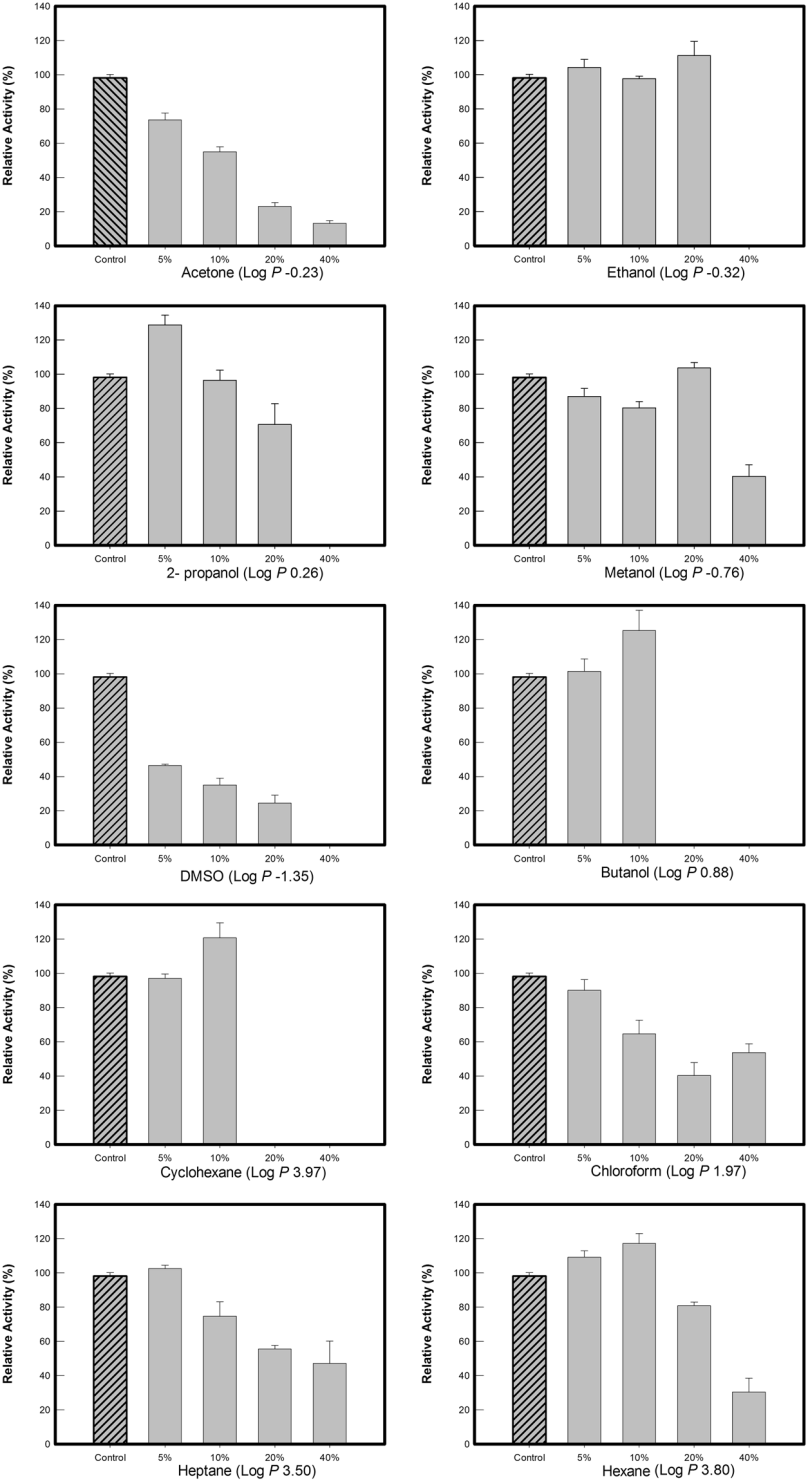
The effect of different organic solvents on MesoAmp activity. The remaining enzymatic activity was determined in the aqueous phase without solvent (none) under the standard conditions of the assay. The Log P (logarithm of the partition coefficient of a particular solvent between n-octanol and water) values determine the degree of hydrophobicity of the solvent. Negative values: hydrophilic solvent and positive values: hydrophobic solvent. The data are expressed as Mean ± SD of five independent experiments.

One of the most interesting characteristics of MesoAmp is its stability in the presence of organic solvents, as shown in Fig. 3. Interestingly, the activity of MesoAmp was stable, particularly at 5% and 10% (v/v) concentrations of ethanol, methanol, 2-propanol and hexane, with a slight increase in activity at 20% (v/v) ethanol and methanol. However, at 40% (v/v), the enzyme was completely inactivated. In the presence of acetone, DMSO, chloroform, butanol and heptane at concentrations above 10%, the enzymatic activity decreased by 50% followed by complete inactivation at higher concentrations of these solvents.

### MesoAmp is not required for growth *in vitro* but is necessary for biofilm production and halotolerance

**Figure 4:**
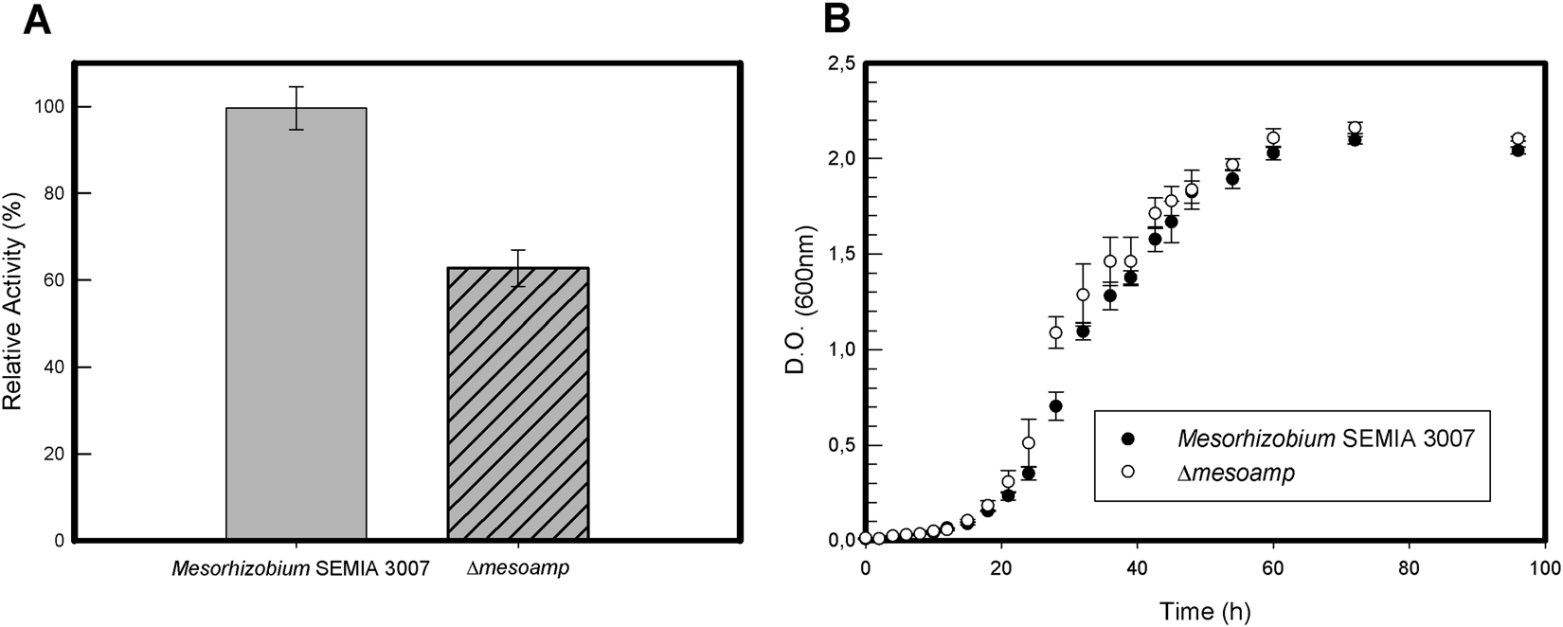
Aminopeptidase activity and *in vitro* growth kinetic assay of *Mesorhizobium* sp SEMIA 3007 and Δ*mesoamp*. (**A**) Enzymatic activity of *Mesorhizobium* sp SEMIA 3007 and Δ*mesoamp* strains using Leu-pNA as the substrate in 100 mM bicarbonate sodium-hydroxide buffer at pH 8.5 and 4 mM Co^2+^. Values are expressed as Mean ± SD. (**B**) *In vitro* growth curves for *Mesorhizobium* sp SEMIA 3007 and Δmesoamp strains. The experiments were performed in triplicate, and the error bars indicate standard errors. The relationship of OD_600_nm to viable count was equivalent for all strains examined. The data are expressed as Mean ± SD of five independent experiments.

MesoAmp was responsible for 40% of the intracellular leucine aminopeptidase activity (Fig. 4A). Nevertheless, both strains *Mesorhizobium* SEMIA3007 and Δ*mesoamp* did not display a significant difference of growth in TY broth (Fig. 4B) suggesting that MesoAmp is not involved in growth in synthetic media. To determine the potential role of peptidases in *Mesorhizobium* SEMIA3007 metabolism, the halo-tolerance, solvent-tolerance and biofilm production of the ∆MesoAmp mutant was evaluated.

**Figure 5:**
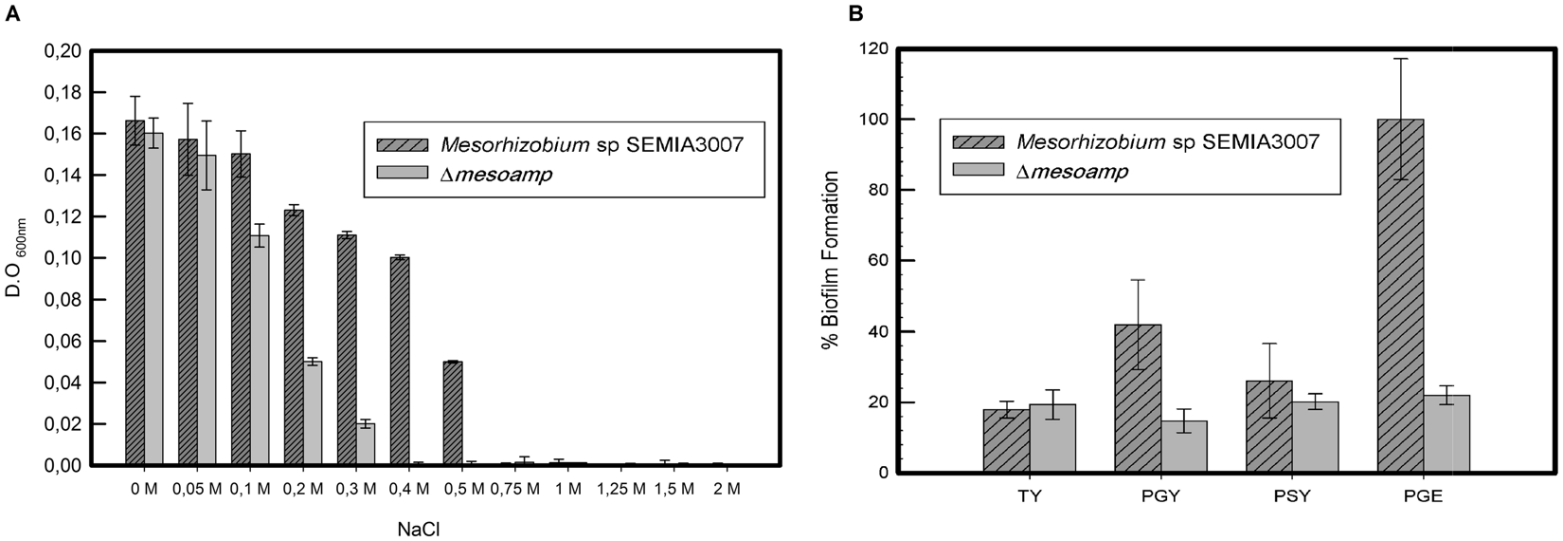
Biofilm production and halotolerance assay. (**A**) Effect of salt on growth of *Mesorhizobium* sp SEMIA 3007 and Δ*mesoamp* strains. Bacteria were inoculated in TY medium containing different salt concentrations. Each point represents the average of three replicates. (**B**) Biofilm formation in *Mesorhizobium* sp SEMIA 3007 and Δmesoamp strains. Each value is mean ± SD; n = 8. Both strains were grown in TY (as control broth), PGY (supplemented with glycerol as carbon source) and PEG (with glucose), and PSY (with sucrose). Biofilm assays were done using precoated plates. The data are expressed as Mean ± SD of five independent experiments.

Biofilm formation is an important microbial survival strategy. We therefore examined whether the absence of the MesoAmp enzyme had an effect on biofilm formation in *Mesorhizobium* SEMIA3007. Figure 5A reveals a significant decrease (80%) in the capacity in biofilm formation in PGE broth (glucose as carbon source) and 50% in PGY broth (glycerol as carbon source) for Δ*mesoamp*. The effect of *mesoamp* gene expression on *Mesorhizobium* stress tolerance was studied by liquid culture assays. When assayed by the liquid culture method under saline stress conditions, growth of Δ*mesoamp* cells decreased significantly with time compared to *Mesorhizobium* SEMIA3007 strain (Fig. 5B). Δ*mesoamp* displayed approximately 60 to 80% decreased saline stress tolerance in 0.2 and 0.3 M of NaCl, respectively, and a total loss of tolerance to higher concentrations of salt (0.4 and 0.5 M, respectively). *Mesorhizobium* SEMIA3007 and Δ*mesoamp* showed tolerance to DMSO and hexane (Figure S5). According to the literature, the mechanisms by which bacteria present solvent tolerance (adaptations such as the efflux pumps, cis-trans fatty acids membrane isomerisation, rapid membrane repair mechanisms and membrane-localized transporters^22, 23^) are several and complexes, thus cannot be attributed to a single gene.

## Discussion

To date, in addition to the genes observed in bacteria, the ones encoding M29 aminopeptidases have been identified in animals, plants and Archea. However, a highly limited number of aminopeptidases M29 from *Thermus aquaticus*, *Thermus thermophilus*, *Bacillus stearothermophilus*, *Streptococcus thermophilus*, *S. pneumoniae*, *S. aureus*, *Borrelia burgdorferi* and *Listeria monocitogenes* have been characterized^13, 20, 24, 25^. In this study, we present the results of genomic data mining in *Mesorhizobium* sp., as well as the cloning, overproduction in *E. coli*, and characterization of a highly halo-tolerant and solvent-tolerant aminopeptidase. This is a promising enzyme with possible applications in the production of debittered hydrolysates, the conversion of L-homophenylalanyl amide into L-homophenylalanine, and the development of flavouring for dairy products^20^.

The protein encoded by the *mesoamp* gene possesses only 40%–50% sequence identity with other members of the M29 family of characterized metallopeptidases. It displays a conserved catalytic domain in the C-terminal region and an 8-element fingerprint that provides a signature for thermophilic metalloproteases (Thermoptase) and virtually spans the full alignment length^26^.

The structural model of MesoAmp displays a long substrate-binding hole in the N-terminal domain that functions to limit the length of the C-terminal side of the substrate. The binding pocket in the C-terminus domain provides the molecular basis for the selectivity of its residues (Fig. 2E)^27^.

The enzymatic characterization of the MesoAmp indicated that the protein retained 80% of its enzymatic activity in an alkaline pH range of 7.5 to 9.0 with maximal activity at pH 8.5. These results are comparable with those on AmpT (optimal pH between 7.0 and 8.0) and PepS aminopeptidase (optimal pH between 7.5–8.5)^28^. According to Wang, T, *et al*.^20^, there is a substantial decrease in the activity outside the pH range 7.0 to 9.0, and it is primarily due to irreversible changes in the protein structure or to the destabilization by buffer species.

The M29 family is characterized by an optimal temperature between 60 to 70 °C^17^. In contrast, MesoAmp had its optimal activity at 40 °C and maintained 80% of its activity at 55 °C with a decrease of 50% of its activity at 60 °C. The thermal stability of proteins seems to be a complex phenomenon that can be affected by many factors. In general, proteins can be stabilized by decreases in their entropy of unfolding^29^. In the primary structure of MesoAmp, the proline residue represents as much as 4.3% of the sequence. At the same time, prolines represent only 5.4% and 4.1% of the residues in the amino acid sequences of Amp T and PePS, respectively. This finding suggests that other factors are responsible for their thermal stability such as the presence of disulfide bonds formed by cysteine residues that stabilize proteins by decreasing the entropy of the protein’s unfolded state^30^ and/or oligomeric state formation, representing an adaptive advantage relative to their monomeric counterparts^24^. The ability to act at temperatures over 40 °C is of great biotechnological importance, reducing the risk of contamination by mesophilic microorganisms. In addition, this characteristic favours substrate and product solubility and increases reaction rates for reducing viscosity and for increasing the diffusion coefficients of the substrates^31^.

Metalloaminopeptidases exhibit a broad range of metal ion dependence, and Zn^2+^ is the most frequently associated cation. Members of the M29 family from *Staphylococcus aureus*, *B. stearothermophilus* and *L. monosytogenes* have Co^2+^ and Mg^2+^ as cofactors. On the other hand, the cofactor for PepS is unknown, but it is unlikely to be Ca^2+^, Cu^2+^, Zn^2+^ Co^2+^, Mn^2+^ and Hg^2+^ because low concentrations of these cations inactivated the enzyme^32^. MesoAmp displays a high dependence on Co^2+^ and Mn^2+^ ions, and the effect of Co^2+^ on aminopeptidases has been frequently described in the literature. This cation increases the relative activity from 3^33^, 5^34^ to 26 times that of the normal activity^35^. A study focusing on density functional theory^36^ determined that the electronic nature of cobalt and manganese ions activates water molecules, thus influencing the generation of the intermediate state (enzyme-substrate), which consequently increases the reaction rate.

MesoAmp exhibited relative resistance in the presence of polar and non-polar solvents, and this phenomenon has been observed in many cases^37^. Nevertheless, the reason for such tolerance has not been established to date. To a certain extent, this solvent activation imparts conformational flexibility to the protein, causing an increase in activity. Additionally, the relative abundance of hydrophobic residues located on the surface of a molecule plays an important role in the structural stability of proteins^37, 38^. Salt has several important effects on the stability of proteins, primarily by charge modification on the protein surface or/and alteration of charges between substrate and enzyme^31^. This effect can subsequently increase enzyme activity. Further studies of the tertiary structure of the aminopeptidase M29 family might clarify the molecular basis of the mechanism of its environmental adaptation at high salt concentrations.

The proteolytic machinery has a functional redundancy between several enzymes. *Mesorhizobium* sp SEMIA3007 produces enzymes with a similar metabolic function (Table S4) according to the Brenda databases^39^. In the absence of MesoAmp, the other enzymes with amino peptidase activity can supply the demand for intracellular amino acids required for bacterial development such as protein synthesis. For that reason, in spite of having a decrease of aminopeptidase activity in the absence of MesoAmp, the growth of *Mesorhizobium* in synthetic media is not affected. However, aminopeptidases may exhibit other functions in addition to the degradation of intracellular peptides and maintenance of the pool of amino acids, such as cell resistance to osmotic stress, biofilm production, and cell invasion^40–42^. To our knowledge, we have characterized aminopeptidase from *Mesorhizobium* or other rhizobacteria for the first time.

Previous work has reported the multifunctionality of aminopeptidases. In *S. typhimurium*, peptidases involved in exogenous peptide degradation, protein turnover and degradation of abnormal proteins^43^. In *E. coli*, PepA is also endowed with DNA binding activity in regulation of the carAB operon^44^. In *Lactococcus lactis*, PepF plays a role in the pyruvate metabolism, cell wall and protein secretion^45^. In *S. thermophilus*, PepS aminopeptidase has cellular functions as peptidoglycan metabolism^12^. Our results suggest that MesoAmp is indispensable for biofilm production and osmotic stress tolerance (Table 4), functions totally undescribed for other aminopeptidases. However, considering that there is no growth defect in the Δ*mesoamp* strain that might account for decreased biofilm production, we suggest that MesoAmp plays a non-nutritional role. The mechanism by which aminopeptidases affect the synthesis of biofilms is not well understood, because their formation and maintenance are multifactorial processes influenced mainly by production and nature of the intra- and extracellular polysaccharides^46^. For *Mesorhizobium*, the fact that MesoAmp contributes to biofilm production might be attributed to the ability of this enzyme to catalyse the peptides to release free leucine and other amino acids that serve as a substrate for a variety of catabolic pathways^47^. In the absence of MesoAmp, the mutant cannot keep that pool of amino acids, thus making it sensitive to osmotic stress. Many species of bacteria have also been shown to increase the proline and leucine pool size and accumulate total free amino acids under osmotic stress contributing to their adaptability to a saline environment^47–49^.

**Table 4:** Biochemical and physiological properties of the M29 family aminopeptidases.

Name	M.O.	Characteristic	Reference
leucine aminopeptidase II	*Bacillus stearothermophilus*	- Homodimer- pH and temperature optimum for the hydrolysis reaction was pH 8.0 and 60 °C.- Co^2+^ ions have a stimulatory effect	76
Aminopeptidase	*Borrelia burgdorferi*	- Homohexameric- pH and temperature optimum for the hydrolysis reaction was pH 7.5 and 60 °C.- Zn^2+^ ions have a stimulatory effect	24
Aminopeptidase T	*Listeria monocytogenes*	- Homodimer- Strongly stimulated by Co^2+^- Virulence Factor for infection	13
Aminopeptidase PepS	*Streptococcus thermophiles*	- Aminopeptidase PepS probably plays a pleiotropic role through its involvement in growth via nitrogen nutrition	12
Alkaline Aminopeptidase	*Bacillus mycoides*	- Monomer- pH optimum for the hydrolysis reaction was 9.0.- Co^2+^ ions have a stimulatory effect- After 30 min preincubation at 45 °C the aminopeptidase retained only 12% of activity.	77
*leucine* aminopeptidase LAP	*Staphylococcus aureus*	- High activity in the presence of Mn^2+^- pH optimum for the hydrolysis reaction was 8.5- broad substrate range that extends beyond leucine	41
Thermostable aminopeptidase	*Aquifex aeolicus*	- pH and temperature optimum for the hydrolysis reaction was pH 8.0 to 8.5 and 80 °C.- Highly resistant to organic solvents.	78
PseA PepB	*Pseudomonas aeruginosa*	- pH and temperature optimum for 6.0 to 8.0 and appreciably thermostable up to 70 °C.- Remarkable stability in both hydrophilic and hydrophobic solvents.	38
BsAmpII	*Bacillus stearothermophilus*	- Homodimer- Active and stable at pHs ranging from 6.5 to 8.5- Secondary structures of BsAmpII are not altered in the presence of 5–10% acetone and ethanol- Remained active at concentrations of urea below 2.7 M	20
Leucine Aminopeptidase	*Bacillus sp*. N2	- Monomeric- pH and temperature optimum for the hydrolysis reaction was pH 9.5 and 50 °C.- Co^2+^ ions have a stimulatory effect.- hydrolytic activity in high concentrations of NaCl (up to 4 M)	79, 80
Mesoamp	*Mesorhizobium SEMIA 3007*	- Homodimer- Strongly stimulated by Co^2+^ and Mn^2+^.- pH and temperature optimum for the hydrolysis reaction was pH 8.5 and 45 °C, and maintained 80% of its activity at 55 °C.- Remarkable stability in organic solvents and was active at high concentrations of NaCl.- Responsible for 40% of the organism’s aminopeptidase activity.- Pleiotropic role through its involvement biofilms formation and halotolerance.	This work

## Conclusions

The major problem of bioprocesses today is the uncompetitive production costs compared to chemical synthesis. High fresh water, energy consuming sterilization, discontinuous fermentation to avoid microbial contamination and the solubility of non-polar substrates^50^ increase production costs. Enzymes with new physicochemical properties (thermo-, solvent- and halo-tolerance) can reduce economic costs, making these enzymes more profitable for industrial bioprocesses. Our bioinformatics approach can greatly shorten the working time^51^ resulting in a significant advantage over conventional methods. Furthermore, in certain cases, along with the metagenomic approach^29^, it is possible to identify new enzymes with high biotechnological potential in a sea of bioinformatics data. Some thermo-tolerant^52^, solvent-tolerant^20^ and halo-tolerant^53^ aminopeptidases have been reported, however, none display all three features simultaneously (see Table 4).

It is very difficult to elucidate the pleiotropic functions of *mesoamp*, mainly due to the complexity of biological systems. We have increased our knowledge of aminopeptidases in the M29 family and confirmed their involvement in the biofilm synthesis and resistance to osmotic stress, but the precise mechanisms by which it interferes with these cellular functions is still unclear. This situation encourages the development of complex and precise biological models that take into account pleiotropy and the connection of all other cellular functions.

## Materials and Methods

The *de novo* sequencing of *Mesorhizobium* SEMIA3007 genome used a combined strategy involving Illumina – HiscanSQ. The prediction of ORFs and annotation were performed using the Rast system^54^ and deposited in the Laboratory of Biochemistry of Microorganisms and Plants (LBMP) database at São Paulo State University, Jaboticabal Campus. The data sets results are available in the NCBI BioProject SRR3703040^55^.

### Homology search and comparative sequence analysis

The annotation of the assembled sequence was conducted using RAST (Rapid Annotation Using Subsystem Technology)^56^. A search for ORFs that encode peptidases was performed, and the selected ORFs were compared individually in the MEROPS peptidase database using the local alignment tool (http://merops.sanger.ac.uk/index.shtml).

### *mesoamp* sequence analysis

Protein signatures and conserved motifs were examined using InterProScan^26^, and the theoretical physiochemical data were generated with the ProtParam tool from ExPASy (http://www.expasy.org/). To estimate the evolutionary conservation of each amino acid translated from the *mesoamp* gene, its sequence was aligned with 24 amino acid sequence from the aminopeptidase members of the M29 family (Table S1) using the CLUSTAL W programme^57^. The alignment was analysed with the ConSurf server^58^. Additionally, an analysis was performed with the SignalP 4.1 server^59^ in order to identify possible signal peptides.

### Molecular modelling

A search for proteins structurally similar to MesoAmp was performed using BLASTP against the Protein Data Bank (PDB). The molecular models of the enzyme were built using the Modeller 9.10 programme^60^ with the default parameters based on the structural coordinates of the aminopeptidase PepS from *Streptococcus pneumoniae* (PDB code: 4ICQ)^27^, the aminopeptidase T from *Thermus thermophilus* (PDB code: 2AYI)^61^ and the aminopeptidase S from *Staphyloccus aureus* (PDB code: 1ZJC)^62^. A quality assessment of the models was performed by ModFold^63^. The scores of the amino acid position conservation displayed by the Consurf server were projected onto the MesoAmp structure. The molecular docking was calculated using M-ZDOCK^64^. The cavity volumes were calculated by KVfinder^65^, and the PyMOL software^66^ was used to visualize the models and to prepare the figures.

### DNA cloning, heterologous overexpression and protein purification

Preparation of plasmids, DNA manipulations, and transformation of *E. coli* BL21(DE3) competent cells were performed as previously described^67, 68^ using the following synthetic oligonucleotides: Forward 5′ CAGGCATATGATCATGACCACACATTCG 3′ and Reverse 5′ CGAACTCGAGCCCTCAGGCCCACT 3′ that had sites for the restriction enzymes NdeI and XhoI, respectively (underlined). The protein concentration was determined using spectrophotometer analysis with a Nanodrop ND-1000 with the theoretical extinction coefficient (54680 M^−1^ cm^−1^) obtained from the ProtParam tool and the Bradford method^69^ with bovine serum albumin (BSA) as a reference.

### Size exclusion chromatography for molecular mass determination

The protein samples were analysed using 10% sodium dodecyl sulfate polyacrylamide gel electrophoresis (SDS-PAGE). The gel was stained with Coomassie Brilliant Blue G-250 and destained with 10% (v/v) acetic acid^70^. The molecular mass of the native enzyme was estimated by size exclusion chromatography (SEC) using a Hiload16/600 Superdex 200 column (GE Healthcare Bio-Sciences, Uppsala, Sweden) previously equilibrated with purification buffer (100 mM Tris-HCl pH 8.0; 200 mM NaCl; 5% (w/v) glycerol). The calibration curve was obtained using the Protein Standard Mix 15–600 kDa (Sigma-Aldrich, St. Louis, MO.), which contains standard proteins [thyroglobulin bovine (670 kDa); γ-globulins (150 kDa); Albumin (44.3 kDa); Ribonuclease A (13.7 kDa); and P-aminobenzoic acid (pABA) (0.13 kDa)], and then plotting the log of the molecular weight of the protein against the elution volume (ml). From this calibration curve, the molecular weight of MesoAmp was determined. The chromatograms were obtained by measuring the absorbance at 280 nm using an ÄKTA pure system (GE Healthcare) at a flow rate of 0.5 mL min^−1^. All collected fractions were analysed by SDS-PAGE, and the fraction containing pure MesoAmp was used for further assays.

### Kinetic assays

Aminopeptidase activity was measured by spectrophotometric detection via the hydrolysis of L-leucine-p-nitroanilide (Leu-p-NA)^20^ by continuously monitoring the p-nitrophenol (p-NA) release at 405 nm using the spectrophotometer reader MultiScan Go (Thermo Scientific). The molar extinction coefficient (εpNA = 9,620) was used to calculate the enzymatic activity. Unless otherwise indicated, the reaction was performed using 1 µg of pure MesoAmp in 20 mM Tris-HCl pH 8.0 buffer at room temperature in a final volume of 200 μL. The assays were carried out in triplicate, and a control was performed for each reaction without the enzyme to measure the spontaneous hydrolysis of the substrates. Linear regressions were performed to determine the initial velocity (Vo) of the reaction.

### Effect of pH and temperature on enzymatic activity

The effect of pH on MesoAmp activity was determined at 35 °C using the following buffers at 100 mM: McIlvaine (pH 3.0 to 4.5); sodium citrate (pH 4.0 to 6.5); sodium phosphate (6.5 to 8.0); Tris-HCl (pH 7.0 to 8.5); bicarbonate-sodium hydroxide (8.5 to 11.0); glycine-sodium hydroxide (8.5 to 10.5) and monosodium phosphate - sodium hydroxide (11.0 to 12.0). The optimal temperature was determined in the range of 10 to 90 °C using 5 °C or 10 °C steps. After these preliminary experiments, the assays were carried out in 100 mM Tris-HCl buffer pH 8.5 at 40 °C.

To verify the thermostability of MesoAmp, an activity assay was performed under the temperature range of 40 °C to 70 °C by pre-incubating the purified enzyme for 5, 15, 30, 45 or 60 minutes followed by an immediate ice bath at 4 °C for 10 minutes. The residual activity was determined after each incubation time.

### Effect of metal ions, peptidase inhibitor agents and detergents on the enzymatic activity

To evaluate the influence of metal ions on MesoAmp activity, 2 mM (final concentration) of K^+^, Mg^2+^, Ca^2+^, Mn^2+^, Co^2+^, Cu^+2^, Fe^+2^, Ni^+2^, Al^+3^, Li^+^, Zn^2+^, Hg^2+^, Cd^2+^ or Ba^2+^ was added to the enzyme solution, and the residual activity was measured. A control without metal ions was used to calculate the residual activity.

Specific peptidase inhibitors were added to the enzyme solution (10 mM) including phenylmethylsulfonyl fluoride (PMSF) or iodoacetic acid that inhibit serine protease and cysteine protease, respectively, and ethylenediamine tetraacetic acid (EDTA) (5, 10 and 20 mM), that inhibits metalloproteases.

The effects of detergents on MesoAmp activity was investigated using Tween 20, Tween 80, Triton X-100 and Triton X114 at 0.5%, 1% and 2% (w/v). The enzyme was incubated with each detergent at room temperature for 30 min. The residual activity was determined and compared to the control. The procedure for the analysis of the effects of ionic surfactants was the same as above except that the enzyme was treated with sodium dodecyl sulfate (SDS) or hexadecyl trimethyl ammonium bromide (CTAB) at 1%, 2% and 4% (w/v).

### Effect of denaturing agents and organic solvents on the enzymatic activity

The effects of urea, guanidine (0.5, 1 and 2 M), NaCl (0.5, 1, 2, 3 and 4 M), Dithiothreitol (DTT) and β-mercaptoethanol (1, 5 and 10 mM) on the activity of MesoAmp were investigated. Samples were incubated at room temperature for 45 min, and the residual activity was determined and compared to the control.

The stability of MesoAmp in the presence of organic solvents was assessed by measuring the residual activity of the enzyme after incubating it at 4 °C for 45 min with 5, 10, 20, and 40% (v/v) of methanol, ethanol, 2- propanol, acetone, chloroform, butanol, cyclohexane, hexane, heptane or dimethyl sulfoxide (DMSO). The resulting activity was compared to the control.

### Kinetic parameters of purified MesoAmp

The K_m_, V_max_ and K_cat_ parameters were calculated from the initial rates of the hydrolysis of Leu-p-NA in the concentration range of 0.05 to 2.5 mM at the optimal conditions for the enzyme, and the non-linear regression of the data using the Michaelis-Menten equation was performed by GraphPad Prism, Version 5.00 for Windows (GraphPad Software, San Diego California USA, www.graphpad.com).

All data obtained were analysed using the R software. An ANOVA and Tukey’s test at 5% probability were used to compare the treatment methods.

### Circular dichroism and thermostability measurements

The circular dichroism (CD) analysis of the purified MesoAmp was performed using a JASCO J-815 spectropolarimeter (Jasco, Tokyo, Japan) linked to a PFD-425S Peltier temperature controller. The enzyme was analysed at 4.4 µmol in 5 mM sodium phosphate, pH 8.0, in the presence and absence of 8.8 µmol Co^2+^. The average of thirty consecutive scans was recorded over a wavelength range of 200 to 260 nm using a 1 mm cuvette, and for both assays, the buffer baseline was subtracted. The thermostability of the enzyme was verified, and its thermal unfolding was monitored by the changes in ellipticity at 222 nm. The temperature was increased with a heating rate of 1 °C min-1 in the range of 20 to 105 °C. The melting point (Tm) represents the temperature at the midpoint of the unfolding transition^71^.

### Construction of *mesoamp* deletion mutant

To generate the Δ*mesoamp* allelic replacement plasmid, 510 and 543 bp DNA fragments located upstream and downstream of the *mesoamp* open reading frame were amplified by PCR and cloned into the nonreplicating vector pNPTS138^72^. The upstream DNA fragment was amplified using primers P(A)F - 5′ TGCGGATCCTGGCGCAGGAAGTGTT 3′ (BamHI restriction sites are underlined), and P(B)R - 5′ CCTCCCTACGTGGGAATCTGACCCATA 3′. The downstream DNA fragment was PCR amplified using primers P(C)F – 5′ GATTCCCACGTAGGGAGGTCGAATGGC 3′ and P(D)R – 5′ CTGGAATTCGTCGGGGACCGGGTTCTG 3′ (EcoRI restriction sites are underlined). A stable in-frame deletion of *mesoamp* was generated by homologous recombination and allelic replacement using an SOE-PCR. The DNA fragment, framed by BamHI and EcoRI sites, was cloned into a BamHI and EcoRI-digested pNPTS138 to generate plasmid pJL38Δ*mesoamp*. *Mesorhizobium* SEMIA3007 electro-competent cells, and electroporation was carried out using the conventional method^73^. Transformant cells with pJL38Δ*mesoamp* integrated upstream of the *mesoamp* locus were obtained by selecting for kanamycin resistance. The recombinants were identified as kanamycin-sensitive colonies and confirmed by PCR with primers P(A)F and P(D)R. The resultant in-frame deletion mutant was further verified by DNA sequencing and finally designated Δ*mesoamp* strain.

### Growth, biofilm production and halotolerance assay

To determine the potential role of aminopeptidases in *Mesorhizobium* sp physiology, Δ*mesoamp* mutant was evaluated in conditions of osmotic and thermic stress and in biofilm production. For routine growth, TY broth^74^ was used. The initial OD_600nm_ of each bacterial suspension was adjusted to 0.05 as a starting time point (0 h). The bacterial cultures were incubated at 30 °C with shaking at 200 rpm and kinetic growth was measured (_OD600 nm_) at 2 h interval for 96 hours.

For comparative analyses of the biofilm production by the wild-type and mutant strains TY, PSY (K_2_HPO_4_ 1.4 g^−l^, KH_2_PO_4_ 1.0 g^−l^, MgSO_4_ 0.2 g^−l^, sucrose 10 g^−l^, yeast extract 1.0 g^−l^), PGY (K_2_HPO_4_ 1.4 g^−l^, KH_2_PO_4_ 1.0 g^−l^, MgSO_4_ 0.2 g^−l^, glycerol 10 g^−l^, yeast extract 1.0 g^−l^) and PGE (K_2_HPO_4_ 0.5 g^−l^, MgSO_4_ 0.2 g^−l^, NaCl 0.1 g^−l^, yeast extract 3.0 g^−l^ and glucose 10.0 g^−l^) broth were used, following incubation at 30 °C with shaking at 200 rpm for 144 h. Then, the biofilms were stained by crystal violet methods as previously described^75^. To investigate halotolerance, *Mesorhizobium* SEMIA3007 and Δ*mesoamp* strain were inoculated into TY broth for 24 h at 30 °C with constant shaking at 200 rpm until the exponential growth phase was reached. Then, the density was estimated spectrophotometrically to achieve an approximate starting concentration (OD_600nm_ = 0.5). The bacterial cultures were incubated into TY broth containing 0.05, 0.1, 0.2, 0.3, 0.4, 0.5, 0.75, 1.0; 1.25, 1.50 or 2 M of NaCl, at 30 °C with shaking at 200 rpm and the OD_600nm_ was monitored for 72 h every twenty-four hours.

### Nucleotide sequence accession number

The DNA sequence of *mesoamp* gene was deposited at GenBank with the reference code WP_069093355.1.

## Electronic supplementary material

Supplementary information
