# Supplementary material for: Halotolerant aminopeptidase M29 from Mesorhizobium SEMIA 3007 with biotechnological potential and its impact on biofilm synthesis
Source: Sci Rep. 2017 Sep 6;7:10684. doi: 10.1038/s41598-017-10932-8 (PMC5587760; doi:10.1038/s41598-017-10932-8)
Supplement: Supplementary file 1 — Supplementary information [file 41598_2017_10932_MOESM1_ESM.pdf]

Halotolerant aminopeptidase M29 from *Mesorhizobium* SEMIA 3007 with biotechnological potential and its impact on biofilm synthesis

Elwi Machado Sierra[1,3]; Mariana Rangel Pereira[2]; Thaís Carvalho Maester[2]; Elisangela Soares Gomes-Pepe[1,2]; Elkin Rodas Mendoza[1]; Eliana de Macedo Lemos[1,2,*,#].

Supplementary information

Table S1 Aminopeptidases M29 family used in the ConSurf analysis

MEROPS Accession	Name	Subfamily	Microorganism
MER001285	aminopeptidase T	M29.001	*Thermus aquaticus*
MER001286	aminopeptidase T	M29.001	*Thermus thermophilus*
MER240296	aminopeptidase T	M29.001	*Deinococcus proteolyticus*
MER346091	aminopeptidase T	M29.001	*Bartonella birtlesii*
MER001287	aminopeptidase II	M29.002	*Geobacillus stearothermophilus*
MER127244	aminopeptidase II	M29.002	*Atopobium parvulum*
MER100581	aminopeptidase II	M29.002	*Bacillus sp.* SG-1
MER002700	aminopeptidase II	M29.002	*Clostridium beijerinckii*
MER062356	PepS aminopeptidase	M29.004	*Bacillus amyloliquefaciens*
MER294030	PepS aminopeptidase	M29.004	*Acetobacter tropicalis*
MER294038	PepS aminopeptidase	M29.004	*Bradyrhizobium sp.* ORS 375
MER251940	PepS aminopeptidase	M29.004	*Geobacillus thermoglucosidasius*
MER118867	aminopeptidase S	M29.005	*Acholeplasma laidlawii*
MER346118	aminopeptidase S	M29.005	*Bacillus macauensis*
MER141311	aminopeptidase S	M29.005	*Clostridium butyricum*
MER160371	aminopeptidase S	M29.005	*Staphylococcus carnosus*
MER346205	non-peptidase homologues	M29.UNW	*Caldicellulosiruptor kristjanssonii*
MER182494	non-peptidase homologues	M29.UNW	*Thermococcus sibiricus*
MER107756	non-peptidase homologues	M29.UNW	*Thermodesulfovibrio yellowstonii*
MER258687	non-peptidase homologues	M29.UNW	*uncultured bacterium*
MER259147	unassigned peptidases	M29.UPW	*Thermacetogenium phaeum*
MER258311	unassigned peptidases	M29.UPW	*uncultured bacterium*
MER294286	unassigned peptidases	M29.UPW	*uncultured Desulfobacterium sp.*
MER142019	unassigned peptidases	M29.UPW	*Verrucomicrobiae bacterium* DG1235

Table S2 Peptidases from *Mesorhizobium* sp

Name	MEROPS Accession Number	Percent Identity in *Mesorizhobium* genome	E-valeu	Family/Clan	homologous peptidase - Microorganism	Catalytic type
Subfamily A24A unassigned peptidases	MER427002	64.74%	8.80e-48	A24/AD	Type IV Prepilin Peptidase - *Pseudomona aeruginosa*	Aspartic
Subfamily A24A unassigned peptidases	MER017898	60.37%	9.00e-46	A24A/AD	Type 4 prepilin peptidase 1 - *Pseudomonas aeruginosa*	Aspartic
Family A24 unassigned peptidases	MER052114	84.09%	5.60e-53	A24X/AD	Type 4 prepilin peptidase 1 - *Pseudomonas aeruginosa*	Aspartic
Family A8 unassigned peptidases	MER014360	97.64%	2.30e-159	A26/AF	Leo peptidase - *Legionella pneumophila*	Aspartic
Family A26 unassigned peptidases	MER014377	88,75%	1.80e-72	A8/AC	signal peptidase II - *Escherichia coli*	Aspartic
Family C14 non-peptidase homologues	MER031247	86.84%	6.40e-116	C14X/	Family C14 non-peptidase homologues	Cystein
Family C82 unassigned peptidases	MER463229	94.7%	5.10e-68	C82/CL	L,D-transpeptidase - *Enterococcus-type*	Cystein
Family I1 unassigned peptidases inhibitors	MER234808	92.31%	1.80e-17	I1	Agalychnis callidryas Kazal trypsin inhibitor	Inibidor
Family I87 unassigned peptidases inhibitors	MER245604	59.3%	7.80e-79	I87	HflC - *Klebsiella sp.*	Inibidor
Family I87 unassigned peptidases inhibitors	MER245615	48.2%	4.00e-52	I87	HflC - *Klebsiella sp. 4_1_44FAA*	Inibidor
Family I87 unassigned peptidases inhibitors	MER409374	50.24%	6.50e-52	I87	QmcA protein	Inibidor
Alanyl aminopeptidase	MER234890	89.76%	2.00e-206	M1/MA	alanyl aminopeptidase - *Escherichia coli*	Metalo
VanX D-Ala-D-Ala dipeptidase	MER014384	95.00%	2.30e-72	M15D/MD	zinc D-Ala-D-Ala carboxypeptidase - *Streptomyces albus*	Metalo

YMXG peptidase	MER015261	99.14%	1.10e-122	M16B/ME	pitrilysin - *Escherichia coli*	Metalo
Subfamily M16B non-peptidase homologues	MER026462	96.65%	3.10e-116	M16B/ME	pitrilysin - *Escherichia coli*	Metalo
Mitochondrial processing peptidase beta-subunit	MER014371	99.56%	1.00e-117	M16B/ME	pitrilysin - *Escherichia coli*	Metalo
PepB aminopeptidase	MER016654	86,31%	-	M17/MF	Aminopeptidase PepB - *Escherichia coli*	Metalo
Similar to cytosol aminopeptidase	MER251584	86.49%	5.10e-194	M17/MF	similar to cytosol aminopeptidase - *Rattus norvegicus*	Metalo
Cysteinylglycinase	MER016655	87.11%	7.60e-216	M17/MF	cysteinylglycinase - *Treponema denticola*	Metalo
Dipeptidase AC	MER014373	94.6%	2.60e-174	M19/MJ	dipeptidase AC - *Acinetobacter calcoaceticus*	Metalo
Mername-AA292 peptidase	MER024185	92%	1.30e-85	M23/MO	Beta-lytic metallopeptidase - *Achromobacter lyticus*	Metalo
DipM g.p.	MER251337	91.82%	4.90e-102	M23B/MO	lysostaphin - *Staphylococcus simulans*	Metalo
Mername-AA292 peptidase	MER024195	98.27%	2.00e-89	M23B/MO	lysostaphin - *Staphylococcus simulans*	Metalo
Mername-AA292 peptidase	MER024183	99.39%	2.60e-87	M23B/MO	Mername-AA292 peptidase - *Neisseria gonorrhoeae*	Metalo
Methionyl aminopeptidase 1 ({Escherichia}-type)	MER014372	99.23%	3.00e-141	M24A/MG	methionyl aminopeptidase 1 - *Escherichia coli*	Metalo
Subfamily M24B non-peptidase homologues	MER026528	91.1%	4.00e-114	M24B/MG	aminopeptidase P - *Escherichia coli*	Metalo
Subfamily M24B non-peptidase homologues	MER251939	99.58%	4.80e-127	M24B/MG	aminopeptidase P - *Escherichia coli*	Metalo
Subfamily M24B non-peptidase homologues	MER026529	93.28%	1.20e-116	M24B/MG	aminopeptidase P - *Escherichia coli*	Metalo
Subfamily M24B non-peptidase homologues	MER026519	99.16%	5.30e-128	M24B/MG	methionyl aminopeptidase 1 - *Escherichia-type*	Metalo
Aminopeptidase P1	MER250815	92.72%	1.10e-267	M24B/MG	methionyl aminopeptidase 1 - *Escherichia coli*	Metalo

Subfamily M24B non-peptidase homologues	MER026541	96.23%	8.90e-126	M24B/MG	subfamily M24B non-peptidase homologues	Metalo
Subfamily M24B non-peptidase homologues	MER026527	95.65%	3.50e-115	M24B/MG	methionyl aminopeptidase 1 - *Escherichia-type*	Metalo
PepS aminopeptidase	MER234460	95.54%	3.20e-208	M29/MQ	PepS g.p. - *Streptococcus thermophilus*	Metalo
Family M32 unassigned peptidases	MER014370	97.16%	1.70e-264	M32/MA	carboxypeptidase Taq - *Thermus aquaticus*	Metalo
Peptidyl-dipeptidase Dcp	MER001902	94.56%	0.00e+00	M3A/MA	thimet oligopeptidase - *Rattus norvegicus*	Metalo
Subfamily M3B unassigned peptidases	MER252922	98.58%	7.60e-152	M3B/MA	oligopeptidase F - *Lactococcus lactis*	Metalo
Subfamily M48B non-peptidase homologues	MER296340	91.8%	4.00e-123	M48B/MA	HtpX peptidase - *Escherichia coli*	Metalo
Subfamily M48C unassigned peptidases	MER026547	93.58%	3.70e-127	M48C/MA	subfamily M48C - unassigned peptidases	Metalo
Family M48 unassigned peptidases	MER026546	92.31%	1.10e-111	M48X/MA	HtpX peptidase - *Escherichia coli*	Metalo
Subfamily M50B unassigned peptidases	MER234855	88.12%	2.50e-162	M50B/MM	Sporulation factor SpoIVFB - *Bacillus subtilis*	Metalo
Murein endopeptidase	MER013983	96.98%	1.10e-145	M74/MD	MepA g.p. - *Escherichia coli*	Metalo
DmpA aminopeptidase	MER510185	48.56%	4.00e-59	P1/PE	DmpA aminopeptidase - *Ochrobactrum anthropi*	Mixed
BapF peptidase	MER500340	48.21%	2.50e-66	P1/PE	DmpA aminopeptidase - *Ochrobactrum anthropi*	Mixed
Pantetheinyl hydrolase ThnT precursor	MER510083	45.57%	1.30e-67	P1/PE	DmpA aminopeptidase - *Ochrobactrum anthropi*	Mixed
Family S11 unassigned peptidases	MER013935	87%	6.40e-171	S11/SE	DacB2 peptidase - *Mycobacterium tuberculosis*	Serine
Family S11 non-peptidase homologues	MER252131	94.69%	1.10e-177	S11/SE	Family S11 unassigned peptidases	Serine
Family S11 unassigned peptidases	MER352127	94.78%	2.30e-111	S11/SE	DacB2 peptidase - *Mycobacterium tuberculosis*	Serine

Family S11 unassigned peptidases	MER013938	98.05%	4.60e-129	S11/SE	D-Ala-D-Ala carboxypeptidase A - *Geobacillus stearothermophilus*	Serine
Peptidase Clp (type 1)	MER014352	98.99%	1.00e-101	S14/SK	Peptidase Clp (type 1) - *Escherichia coli*	Serine
Peptidase Clp (type 1)	MER016653	100%	6.00e-104	S14/SK	peptidase Clp (type 1) - *Escherichia coli*	Serine
Family S14 non-peptidase homologues	MER054907	71.29%	1.90e-77	S14/SK	peptidase Clp (type 1) - *Escherichia coli*	Serine
Lon-A peptidase	MER013944	99.6%	4.60e-129	S16/SJ	Lon-A peptidase - Escherichia *coli*	Serine
Subfamily S1A unassigned peptidases	MER014364	92.2%	5.00e-148	S1A/PA	chymotrypsin A - *Bos taurus*	Serine
Subfamily S1B unassigned peptidases	MER016326	98.56%	2.50e-114	S1B/PA	glutamyl peptidase I - *Staphylococcus aureus*	Serine
Subfamily S1B unassigned peptidases	MER016327	99.11%	2.80e-122	S1B/PA	glutamyl peptidase I - *Staphylococcus aureus*	Serine
Subfamily S1B non-peptidase homologues	MER252019	91.53%	2.70e-117	S1B/PA	exfoliative toxin ExhD - *Staphylococcus hyicus*	Serine
HtrA peptidase	MER234813	97.56%	3.70e-120	S1C/PA	DegP peptidase - *Escherichia coli*	Serine
DegQ peptidase	MER252342	96.65%	1.10e-175	S1C/PA	chymotrypsin A (cattle-type) - *Bos taurus*	Serine
DegQ peptidase	MER250857	86.67%	2.80e-177	S1C/PA	DegP peptidase - *Escherichia coli*	Serine
DegQ peptidase	MER234833	92.33%	8.70e-167	S1C/PA	DegP peptidase - *Escherichia coli*	Serine
DegQ peptidase	MER208157	90.61%	1.90e-155	S1C/PA	DegQ peptidase - *Escherichia coli*	Serine
Repressor LexA	MER013948	100%	7.80e-56	S24/SF	repressor LexA - *Escherichia coli*	Serine
Subfamily S26A unassigned peptidases	MER026322	100%	1.50e-116	S26A/SF	sipC g.p. - *Bacillus caldolyticus*	Serine

Family S33 unassigned peptidases	MER026348	94.35%	2.60e-158	S33/SC	family S33 unassigned peptidases	Serine
Prolyl aminopeptidase	MER013951	97.11%	3.60e-170	S33/SC	prolyl aminopeptidase - *Neisseria gonorrhoeae*	Serine
C-terminal processing peptidase-3	MER250743	99.69%	2.10e-165	S41A/SK	C-terminal processing peptidase-1 - *Escherichia coli*	Serine
Subfamily S49C unassigned peptidases	MER036999	81.77%	1.90e-77	S49C/SK	subfamily S49C unassigned peptidases	Serine
RhoII peptidase	MER026387	86%	1.20e-100	S54/ST	RhoII peptidase - Haloferax volcanii	Serine
Prolyl endopeptidase	MER013931	97.84%	2.50e-146	S9A/SC	prolyl endopeptidase - *Myxococcus xanthus*	Serine
At1g69020 g.p.	MER025184	93.73%	1.60e-144	S9A/SC	At1g69020 g.p. - *Arabidopsis thaliana*	Serine
PfHslV peptidase	MER323710	90.64%	2.70e-76	T1B/PB	HslV component of HslUV peptidase - *Escherichia coli*	Treonin
Gamma-glutamyltransferase 1 (bacterial-type)	MER014380	91.32%	1.60e-268	T3/PB	gamma-glutamyltransferase 1 - *Escherichia coli*	Treonin
Family T3 unassigned peptidases	MER026400	88.78%	6.20e-253	T3/PB	gamma-glutamyltransferase 1 - *Escherichia coli*	Treonin

Table S3. Peptidases with biotechnological applications found in the *Mesorhizobium* SEMIA3007 genome.

Name	Identity (%)	E-value	Peptidase Family	Outstanding physicochemical feature	Biotechnology application
Leucine aminopeptidases from *Burkholderia pseudomallei* (EC 3.4.11.1)	41%	3e-92	M17	Activity from pH 7.0 to 10.5 and optimum temperature of 70 °C [30]	Preparation of debittered hydrolysates and the conversion of l-homophenylalanyl amide into l-homophenylalanine [71]

Carboxypeptidase Taq from *Thermus aquaticus* (EC 3.4.17.19)	39%	1e-104	M32	Activity from pH 6.0 to 7.0 and optimum temperature from 80 to 100 °C [30]	High-temperature analysis of the C-terminal amino acid sequences of proteins [72]
Aminopeptidase T (EC 3.4.11.10) from *Thermus thermophilus.*	54%	1e-107	M29	Activity from pH 7.5 to 8.5 and optimum temperature from 70 to 80 °C [30]	Used for N-terminal sequence determination. Hydrolysis of peptides can affect their bitter taste [30]

Table S4. Aminopeptidases with similar activity of MesoAmp founded in *Mesorhizobium* sp SEMIA3007 genome.

**Name**	**E.C.**	**Family/Clan**	**Recommended Name**	**Catalytic type**
Aminopeptidase P	3.4.11.9	M24B/MG	Xaa-Pro aminopeptidase	Metallo
Alanyl aminopeptidase	3.4.11.14	M1/MA	Cytosol alanyl aminopeptidase	Metallo
Family P01 unassigned peptidase	-	P1/PE	-	Mixed
PepB aminopeptidase	3.4.11.23	M17/MF	PepB aminopeptidase	Metallo
MesoAmp	3.4.11.24	M29/MQ	Aminopeptidase T	Metallo

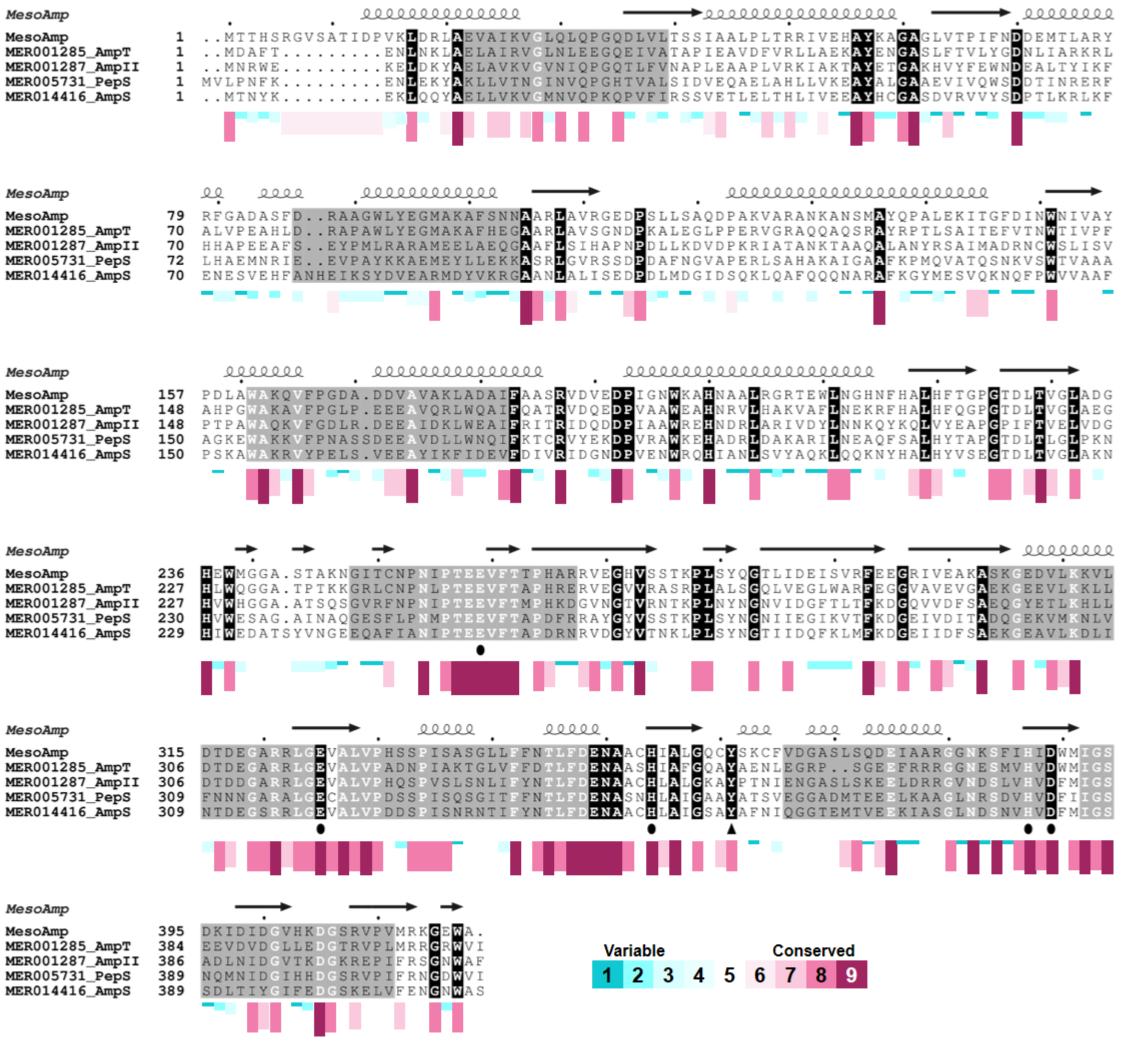

Supplementary Figure S1. Multiple sequence alignment of MesoAmp shows conserved regions when compared to the most representative members of the aminopeptidase M29 family. Amino acid sequence alignment of MesoAmp with aminopeptidase T from *Thermus thermophilus* (MER001285), aminopeptidase II from *Geobacillus stearothermophilus* (MER001287), aminopeptidase S from *Streptococcus thermophilus* (MER005731), and aminopeptidase S from *Staphylococcus aureus* (MER014416) constructed using ClustalX and displayed with Espript 3 (http://espript.ibcp.fr/ESPript/ESPript/). The predicted secondary structure of MesoAmp is shown at the top, with the arrows indicating β-sheet, and the spirals indicatingα helix. The colorful bar on the bottom of the alignment represents the scale of non-conserved (blue) to highly conserved (purple) states for each amino acid of MesoAmp, according to the ConSurf server analysis. The fingerprint element that provides a signature for thermophilic metalloproteases is highlighted in gray boxes. The metal-binding residues

are indicated with a circle, (●) and the catalytic residues are indicated with a triangle (▲).

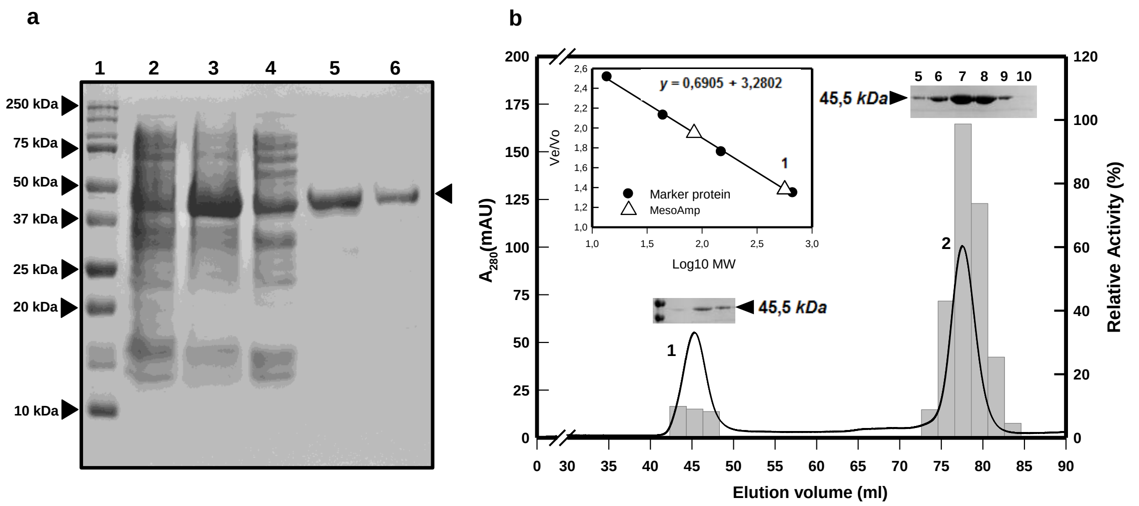

Supplementary Figure S2. The over-expression and purification of MesoAmp. (A) SDS-polyacrylamide gel electrophoresis at each purification step. Lane 1, molecular mass maker; Lane 2, total soluble extract after induction; Lane 3, total soluble extract before induction; Lane 4, flow-through fraction from the Ni-NTA purification; Lane 5, 500 mM imidazole elution fraction; Lane 6, purified aminopeptidases after gel filtration chromatography. The right arrow indicates the molecular mass of MesoAmp. (B) The elution profile of MesoAmp from analytical gel-filtration using Superdex-200. Black line: (▬) mAU, gray bar: relative activity. Peak 1 corresponds to aggregating oligomers of MesoAmp with low activity. Peak 2 is the fractions with the highest activity. The box shows the molecular mass analysis of the native MesoAmp with marker proteins. The SDS-PAGE analysis from gel-filtration chromatography fractions. Lane 1, molecular marker; lanes 2–4; Peak 1; Lanes 6-10; Peak 2.

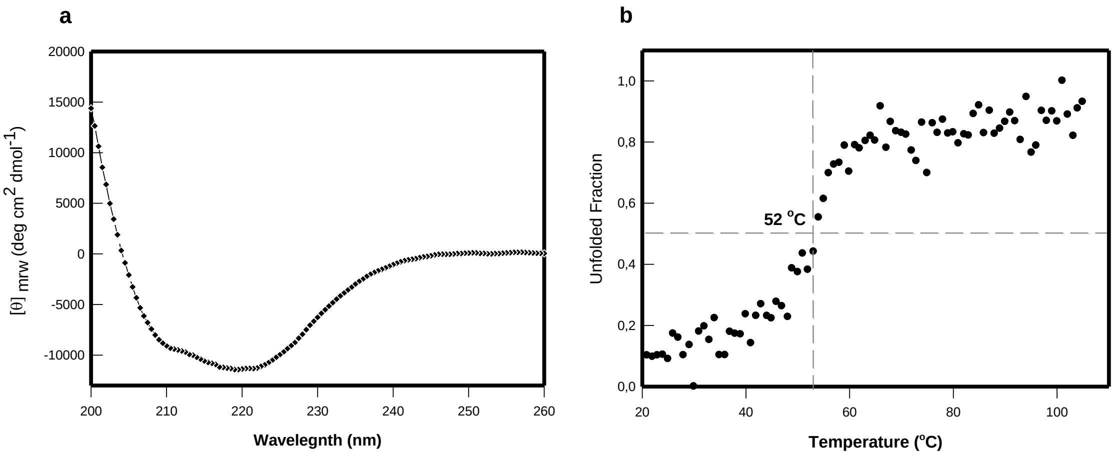

Supplementary Figure S3. The spectroscopic analyses and thermal denaturation of MesoAmp. (A) The circular dichroism spectrum of MesoAmp determined in 100 mM Bicarbonate-Sodium hydroxide buffer (pH 8.5). (B) The therrmal denaturation profiles of MesoAmp. The changes in ellipticity at 222 nm was plotted as a function of the temperature at pH 8.5. The interception at the dotted lines indicates the melting temperature (T_m) estimated by fitting the data using the five-parameter sigmoid function from the curve-fitting program SigmaPlot.

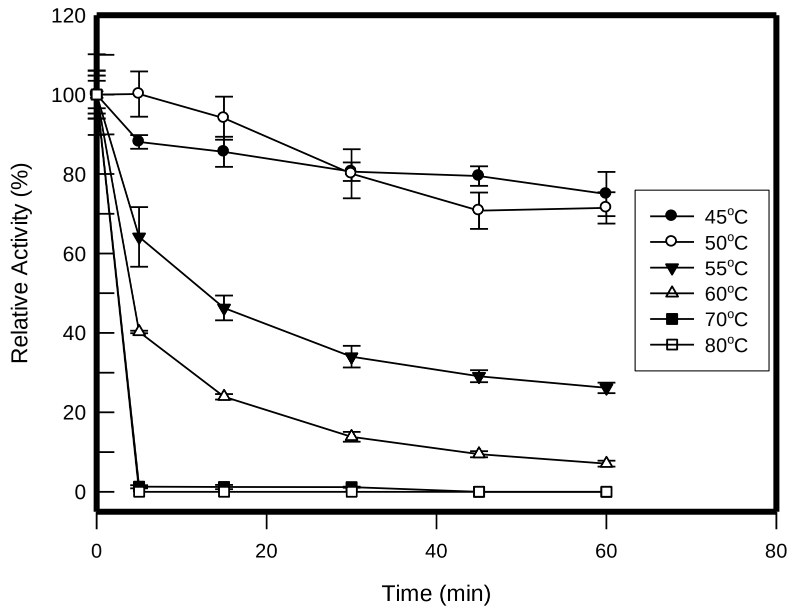

Supplementary Figure S4. Thermostability of MesoAmp using Leu-p-NA as substrate. Assay performed at different temperatures for different times (5, 15, 30, 45, 60 min). The data are expressed as Mean ± SD of five independent experiments.

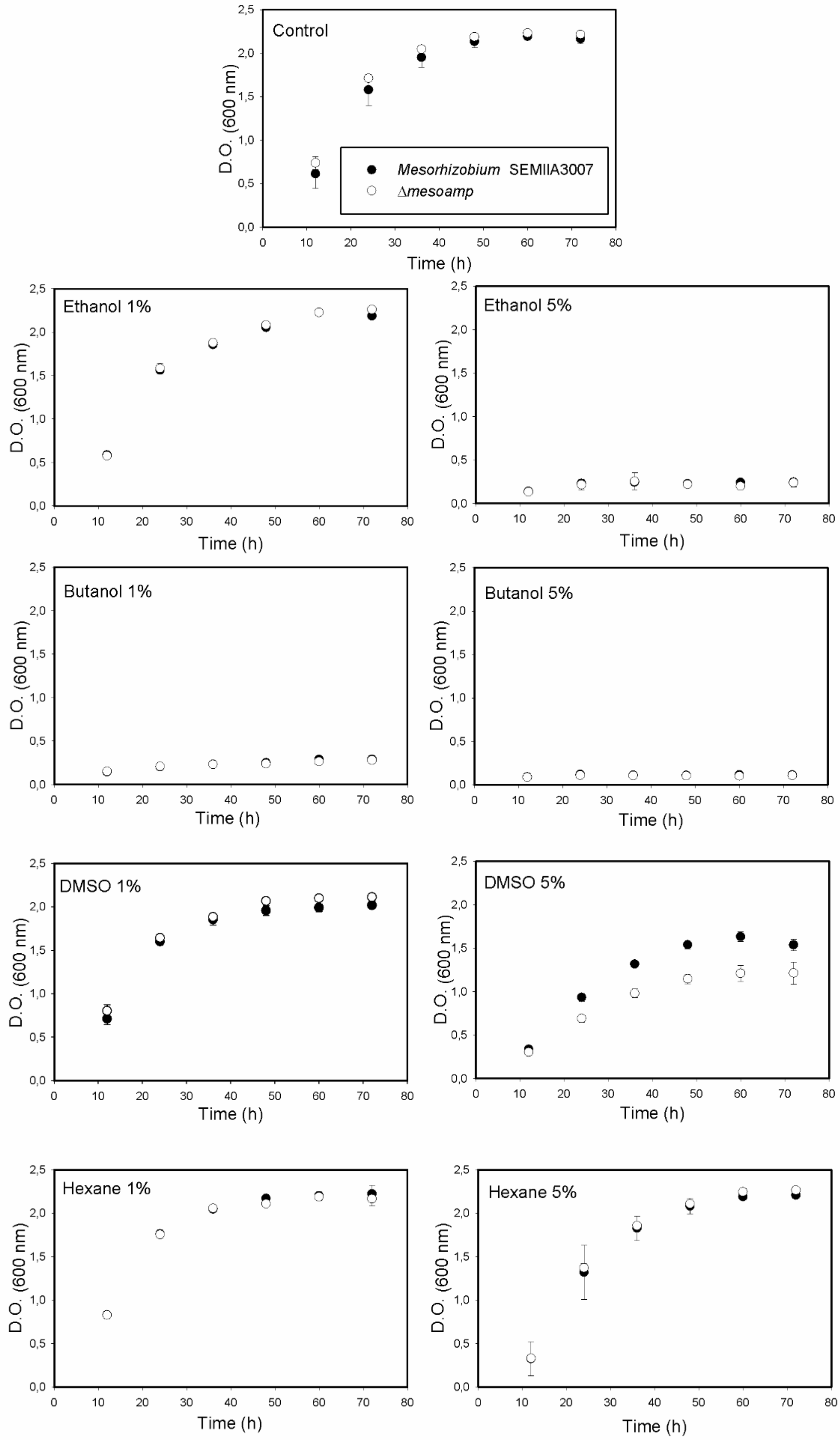

Control
Mesorhizobium SEMIIA3007
Δmesoamp
Ethanol 1%
Ethanol 5%
Butanol 1%
Butanol 5%
DMSO 1%
DMSO 5%
Hexane 1%
Hexane 5%
D.O. (600 nm)
Time (h)

Supplementary Figure S5. Effect of organic solvents on kinetics growth of *Mesorhizobium* SEMIA 3007 and Δ*mesoamp strain*
